# Should First-line Empiric Treatment Strategies for Neonates Cover Coagulase-negative Staphylococcal Infections in Kenya?

**DOI:** 10.1097/INF.0000000000001699

**Published:** 2017-10-13

**Authors:** Anna C. Seale, Christina W. Obiero, Kelsey D. Jones, Hellen C. Barsosio, Johnstone Thitiri, Moses Ngari, Susan Morpeth, Shebe Mohammed, Gregory Fegan, Neema Mturi, James A. Berkley

**Affiliations:** From the * Kenya Medical Research Institute (KEMRI)-Wellcome Trust Research Institution, Kilifi, Kenya; † University of Oxford, Oxford, United Kingdom; and ‡ London School of Hygiene and Tropical Medicine, and § Imperial College London, London, United Kingdom.

**Keywords:** neonatal infection, *Staphylococcus*, contaminant, sepsis

## Abstract

Neonatal mortality remains high in sub-Saharan Africa, and a third of deaths are estimated to result from infection. While coagulase-negative staphylococci (CoNS) are leading neonatal pathogens in resource-rich settings, their role, and the need for early anti-Staphylococcal treatment in empiric antibiotic guidelines, is unknown in sub-Saharan Africa.

**Methods:**

We examined systematic clinical and microbiologic surveillance data from all neonatal admissions to Kilifi County Hospital (1998–2013) to determine associated case fatality and/or prolonged duration of admission associated with CoNS in neonates treated according to standard World Health Organization guidelines.

**Results:**

CoNS was isolated from blood culture in 995 of 9552 (10%) neonates. Case fatality among neonates with CoNS isolated from blood did not differ from other neonatal admissions (*P* = 0.2), and duration of admission was not prolonged [odds ratio (OR) = 0.9 (0.7–1.0), *P* = 0.040]. Neonates with CoNS were more likely to have convulsions [OR = 1.4 (1.0–1.8), *P* = 0.031] but less likely to have impaired consciousness or severe indrawing [OR = 0.8 (0.7–0.9), *P* = 0.025; OR = 0.9 (0.7–1.0), *P* = 0.065].

**Conclusions:**

CoNS isolation in blood cultures at admission was not associated with adverse clinical outcomes in neonates treated according to standard World Health Organization guidelines for hospital care in this setting. There is no evidence that first-line antimicrobial treatment guidelines should be altered to increase cover for CoNS infections in neonates in this setting.

## Abstract 2

Supplemental Digital Content is available in the text.

While advances have been made in reducing global deaths in children under 5 years old, neonatal mortality remains high, with the greatest burden in sub-Saharan Africa. Around a third of neonatal deaths are attributed to infection, and there is also a considerable burden of morbidity because of subsequent disability.^1,2^

Data on the etiology of neonatal infection remain limited,^3^ and improving our understanding of this is essential to guide treatment strategies. Most cases of possible serious bacterial infection (pSBI) in neonates in sub-Saharan Africa are treated empirically according to World Health Organization (WHO) guidelines in the absence of access to microbiologic laboratory facilities. The first-line antimicrobials recommended are amoxicillin and gentamicin, unless Staphylococcal infection is suspected from clinical signs such as skin pustules or abscesses.^4,5^

In resource-rich settings, coagulase-negative Staphylococci (CoNS) have emerged as leading pathogens in certain vulnerable patient groups, including neonates, particularly if they are preterm.^6,7^ This is associated with greater use of indwelling medical devices, such as peripherally inserted central catheters, and ventilators, which CoNS have the ability to colonize through the formation of biofilms.^6,8,9^ CoNS infection also increases morbidity because of chronic lung disease and adverse neurodevelopmental outcomes, as well as being associated with prolonged hospital stay.^10^ However, CoNS is not strongly associated with increased mortality,^11^ compared, for example, with *Staphylococcus aureus* or Gram-negative infections.^12^

In resource-poor settings, such as sub-Saharan Africa, CoNS are variably reported as clinically significant,^13–18^ or not,^19–23^ and in neonates account for 6%–32%^13,15,24^ of all positive blood cultures. Assessing their clinical importance is difficult; they are common skin commensals and can thus contaminate samples. Levels of medical support are usually lower than resource-rich settings,^25^ which may make isolation of CoNS less likely to be clinically significant, but particularly as the proportion of preterm births is high, this is an important question to answer.^26^ There are limited data currently available, with few pathogen-specific mortality outcomes reported.

Data from a tertiary care unit in South Africa reported low case fatality (2%),^27^ but data from lower level health facilities are lacking.

This study therefore aimed to examine whether CoNS isolated in blood cultures from neonates admitted to a rural hospital in Kenya, treated according to standard WHO guidelines, were associated with death, prolonged hospital duration or specific clinical features of pSBI. To do this, the specific objectives were to test the association between death, prolonged hospital duration or features of pSBI in the following:

1. Neonates with CoNS isolated from blood cultures compared with all other admissions (primary analysis).
2. Neonates with CoNS isolated from blood cultures compared with other admissions excluding those with clinically significant organisms isolated.
3. Neonates with CoNS isolated from blood cultures compared with neonates with clinically significant isolates in blood cultures.
4. Neonates with clinically significant isolates in blood cultures compared with all other admissions.
5. Neonates with clinically nonsignificant or uncertain significance isolates (excluding CoNS) compared with all other admissions.

## METHODS

### Study Design and Setting

This retrospective cohort study was undertaken using systematic clinical and microbiologic surveillance data from Kilifi County Hospital (KCH). KCH serves Kilifi County, which had a population of 540,000 in 2009, and covers a large catchment area (12,246 km^2^). The main local economy is subsistence farming,^28^ and nearly 3 quarters of the population live below the nationally defined poverty line.^29^ KCH provides pediatric care with ~4000 admissions each year and comprehensive maternity care for ~3000 deliveries each year. It serves as a referral center for peripheral health facilities.

### Recruitment

All neonates (0–27 days) admitted to the hospital between August 1, 1998, and December 31, 2013 were included in this study, unless admitted for elective procedures. The sample size was determined by the number of admissions during the surveillance period.

### Clinical Investigation

Systematic clinical admission data were collected on all neonatal admissions and prospectively entered into databases. This included the presence of signs of pSBI, as defined by the Young Infants Clinical Signs Study,^30^ including difficulty feeding, convulsions, reduced conscious level, hypothermia or fever, high respiratory rate, movement only when stimulated and chest wall in-drawing. Other measurements included anthropometry (admission weight) and routine clinical investigations (hematology, biochemistry and blood culture) for all neonatal admissions. Cerebrospinal fluid (CSF) was sampled where there was no contraindication; however, CoNS were not systematically reported in CSF. Since 2007, HIV testing has been offered routinely, in line with national guidelines for provider- initiated testing in Kenya.

At KCH, neonates are treated according to standard protocols, based on WHO guidelines.^4,31^ These include intravenous ampicillin and gentamicin as first-line antimicrobials,^4^ and usually a third generation cephalosporin as second line. KCH was reliably supplied with intravenous cannulas, first- and second-line empiric antibiotics and oxygen during the study period. Medical officers were trained in neonatal resuscitation, but neonatal ventilation was not available and peripherally inserted central catheters were not in use. No specific treatment was initiated as a result of isolating CoNS from blood and species identification, and antimicrobial susceptibility testing were not performed.

### Sampling

Clinical procedures have been described previously.^21^ Briefly, for blood cultures, an aseptic technique was used and blood was inoculated into a blood culture bottle (BACTEC Peds Plus; Becton Dickinson, Franklin Lakes, NJ). Blood culture bottles were weighed before and after inoculation and processed with an automated blood culture system (BACTEC 9050; Becton Dickinson). Positive blood cultures were cultured on standard media. CoNS were reported, but species were not determined, and CoNS isolates were not stored or tested for antibiotic susceptibilities, as they were not regarded as clinically significant. All laboratory procedures were internally controlled, and the KEMRI-Wellcome Trust Research Laboratories were externally monitored for quality assurance by the United Kingdom National External Quality Assessment Service and were Good Clinical Laboratory Practice–accredited by Qualogy, United Kingdom, since 2007. From 2009, a working group was formed as a quality improvement activity to reinforce training in aseptic technique with oversight and feedback of rates of presumed contaminants.

### Analysis

Clinical data were extracted from databases and merged with laboratory databases using unique numerical identifiers to link data (personal identifiers were removed). Data analysis was performed with STATA version 13 (Stata Corp, College Station, TX). Data were described, and univariable and multivariable logistic regression analyses done for each objective, adjusting in each, for age, sex and volume of blood for culture a priori. The volume of blood for culture was included as a potential confounder a priori because if blood sampling was difficult, a small sample was considered more likely to have been obtained (eg, from a neonate with poor peripheral circulation), and contamination more likely. All clinical signs associated with CoNS isolation in univariable analyses (*P* < 0.1) were included in the multivariable model for each analysis, with backwards stepwise regression based on the likelihood ratio test (LRT) used to determine the final model.

For the first objective, and primary analysis, the unexposed group were all neonates who had a blood culture without CoNS isolated from blood. This analysis was chosen because CoNS can mask the growth of other organisms, and if those with CoNS isolated were compared with only those with no pathogen isolated, increased mortality caused by CoNS could be because of known pathogens being masked by CoNS. However, to investigate differences between children with CoNS and those with other specific blood stream infection categories, other comparisons were made as per objectives 2 and 3, as well as comparing neonates with clinically significant isolates to all others as per objective 4 and neonates with other clinically nonsignificant isolates or isolates of unknown significance from blood culture (*Bacillus* spp., Coryneforms, *Micrococcus* spp. and Viridans-group *Streptococci*) compared with all others, as per objective 5.

#### Ethical Review

This study was approved by KEMRI National Ethical Review Committee, Nairobi, (SSC/ERC 2906). This analysis was a retrospective review of systematically collected routine clinical data and laboratory findings for a neonatal admissions to KCH.

## RESULTS

### Participants

There were 10,058 neonates admitted to KCH between the August 1, 1998, and December 31, 2013. Of these, 15 (0.1%) were ineligible as they were admitted for elective surgery. A further 115 (1.1%) were eligible but excluded from the analysis as outcome data were missing. There were 376 (3.7%) neonates who did not have a blood culture, leaving 9552 in the analysis.

### Bacterial Isolates

Of 9552 neonates, 995 (10.4%) had CoNS isolated from blood culture, of which 150 of 995 (15.1%) died (Table 1). There were 689 of 9552 (7.2%) neonates with clinically significant isolates, of whom 241 of 689 (35.0%) died (Table S1, Supplemental Digital Content 1, http://links.lww.com/INF/C774). There were 587 of 9552 (6.1%) neonates with clinically nonsignificant pathogens or isolates of unknown significance from blood culture in this context (*Streptococcus viridans*, Coryneforms, *Micrococcos* spp. and *Bacillus* spp.), 124/587 (21.0%) died. Of all positive cultures, CoNS accounted for 995 of 2271 (43.8%) and (983/995, 98.8%) were sampled in the first 48 hours of admission. Overall mortality was 1873 of 9552 (19.6%).

**TABLE 1.**
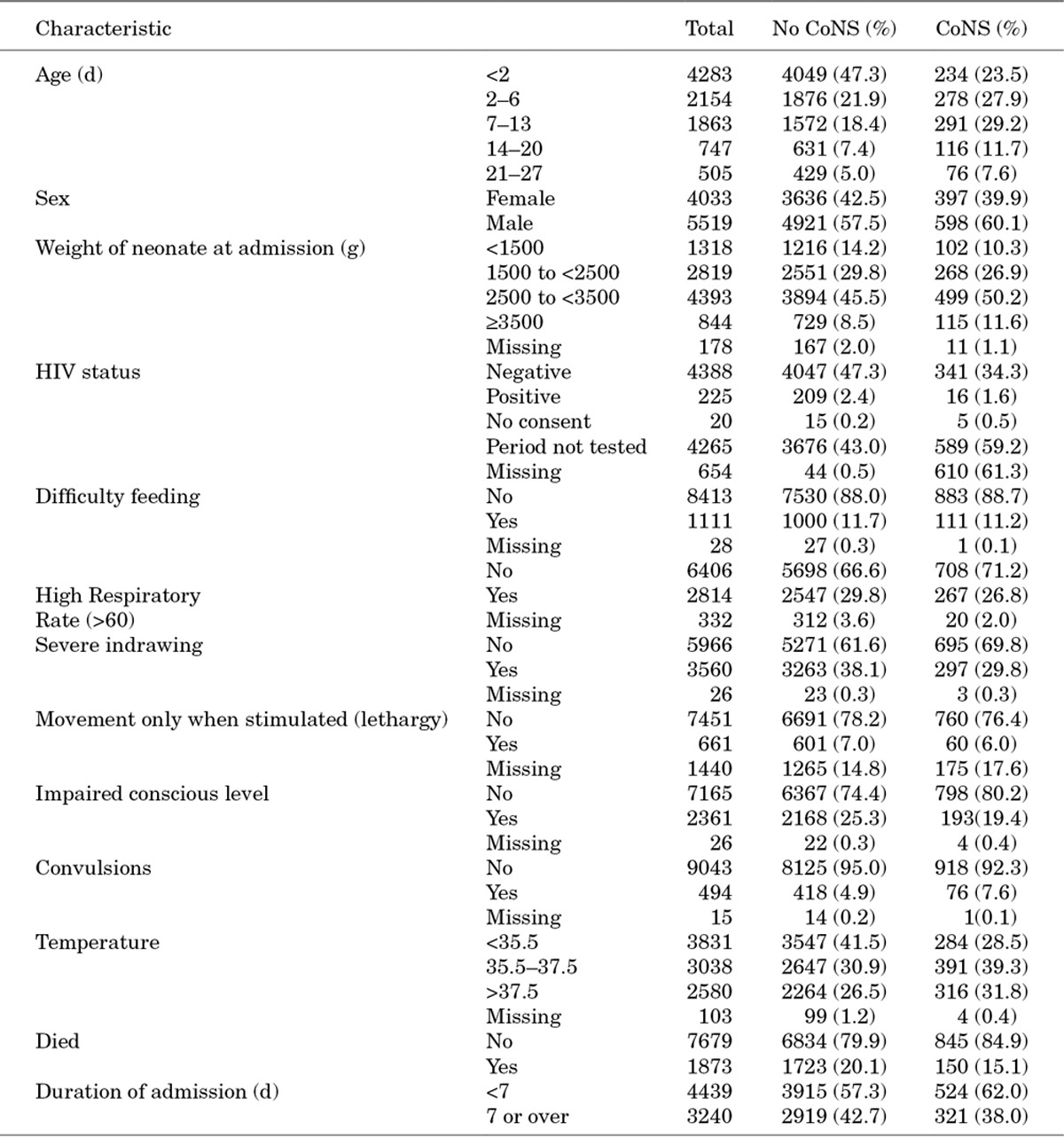
Demographic and Clinical Characteristics of Neonates Admitted to Kilifi County Hospital and Those With CoNS Isolated From Blood Sampling

**FIGURE 1.**
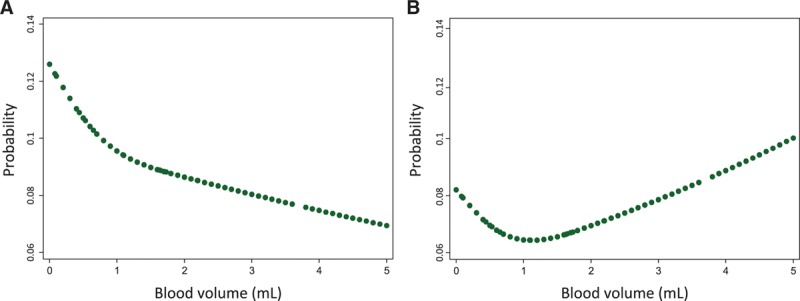
A: Probability of isolating CoNS from blood culture against the volume of blood in the culture. B: Probability of clinically significant isolate against blood culture volume.

The probability of isolating CoNS decreased with increasing blood culture volume [odds ratio (OR) = 0.80 (0.71–0.90), *P* < 0.001 for each mL increase], with no evidence of departure from linearity (LRT, *P* = 0.8), Figure 1A, while clinically significant organisms were more commonly isolated from higher blood culture volumes (Fig. 1B). Bacterial isolates are shown for each year in Figure S1, Supplemental Digital Content 1, http://links.lww.com/INF/C774, with reductions in isolations of CoNS after the introduction of a blood culture contamination working group which reinforced training for hospital staff on using stringent aseptic techniques for blood culture collection.

#### 1. Neonates With CoNS Isolated From Blood Cultures Compared With all Other Admissions (Primary Analysis)

Isolation of CoNS in neonates was not associated with inpatient death [OR = 0.9 (0.7–1.0), *P* = 0.1]. It was, however, associated with decreased duration of admission in survivors [OR for admission of 7 days or more = 0.9 (0.7–1.0), *P* = 0.040] when compared with all other admissions.

In terms of clinical signs, CoNS isolation was associated with increased odds of convulsions [OR = 1.4 (1.0–1.8), *P* = 0.031], decreased odds of impaired conscious level and severe indrawing [OR = 0.8 (0.7–0.9), *P* = 0.025; OR = 0.9 (0.7–1.0), *P* = 0.065] compared with all other admissions (Table 2). There was no evidence of an association between CoNS and other clinical signs, admission weight or HIV exposure. With regards to timing of infection, neonates admitted after the first 48 hours of birth were more likely to have CoNS isolated [OR = 2.3 (1.8–2.8), *P* < 0.001] compared with admissions during days 0–2 of life, with evidence of departure from linearity across age categories, suggesting no further increases with increasing age (LRT, *P* < 0.001), Table 2.

**TABLE 2.**
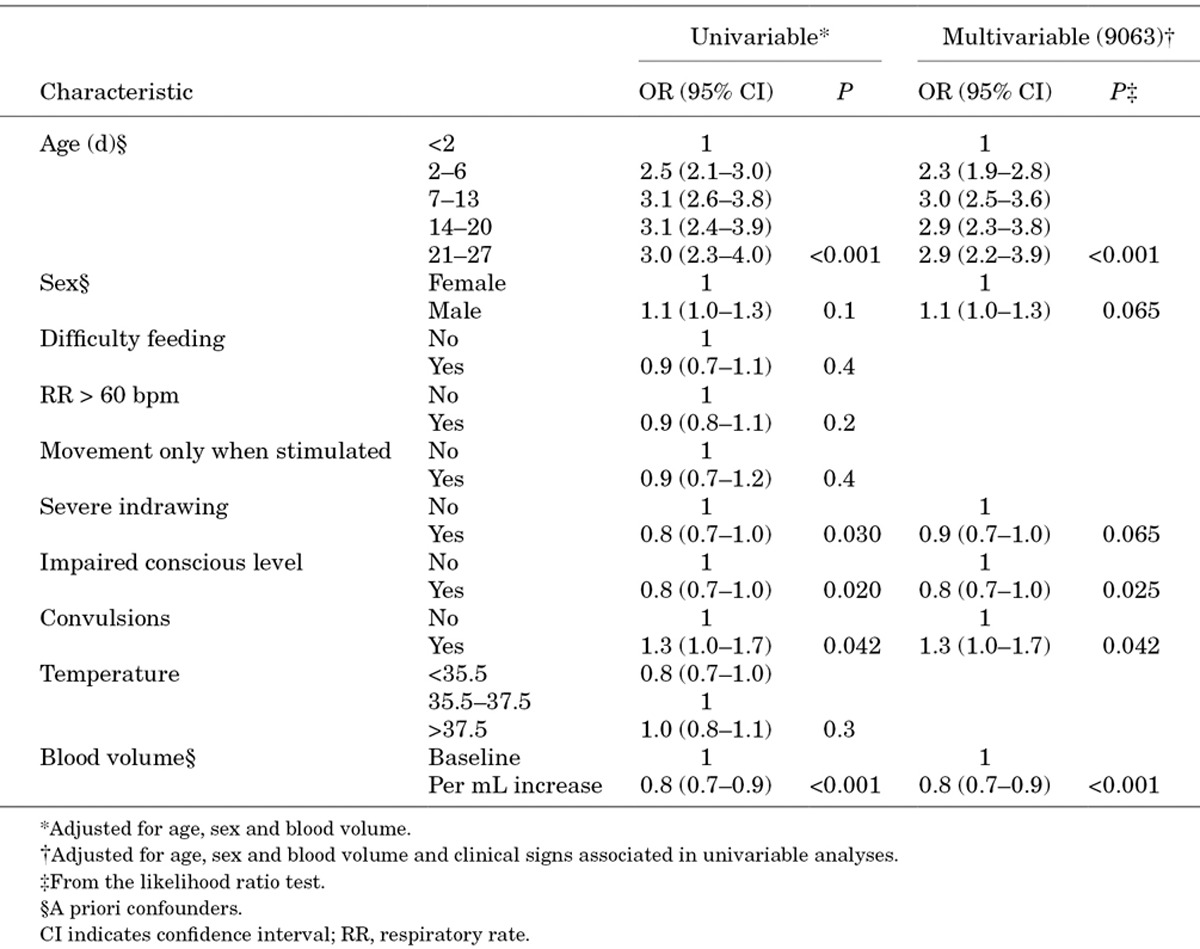
Demographic and Clinical Characteristics Associated With Isolation of Cons From the Blood in Neonates Admitted to Kilifi County Hospital 1998–2013 Compared With all Other Neonatal Admissions

#### 2. Neonates With CoNS Isolated From Blood Cultures Compared With Other Admissions Excluding Those With Clinically Significant Organisms Isolated

Isolation of CoNS in neonates was not associated with inpatient death after excluding neonates with presumed clinically significant bacteremia [OR = 1.0 (0.8– 1.2),*P* = 0.9] nor was it associated with a difference in duration of admission (7 days or more) in survivors after excluding those with clinically significant bacteremia [OR = 0.9 (0.8–1.1),*P* = 0.3]. Clinical associations and timing of infections after exclusion of neonates with clinically significant bacteremia were similar to those described in the primary analysis (Table S2, Supplemental Digital Content 1, http://links.lww.com/INF/C774).

#### 3. Neonates With CoNS Isolated From Blood Cultures Compared With Neonates With Clinically Significant Isolates in Blood Cultures

Isolation of CoNS in neonates was associated with lower mortality compared to those with a clinically significant blood culture isolates [OR death during admission = 0.3 (0.3–0.4), *P* < 0.001] and decreased duration of admission in survivors [OR for admission of 7 days or more = 0.3 (0.3–0.4), *P* < 0.001]. The odds of clinical signs of pSBI were much reduced for all signs except convulsions, for which no difference in association was found and it was not included in the final multivariable model (Table S3, Supplemental Digital Content 1, http://links.lww.com/INF/C774).

#### 4. Neonates With Clinically Significant Isolates in Blood Cultures Compared With all Other Admissions

In neonates with clinically significant bacteremia, compared with all other neonatal admissions, the odds of death were high [OR = 3.1 (2.6–3.7), *P* < 0.001], and there was a prolonged duration of admission [OR for admission 7 days or more in survivors = 1.7 (1.4–2.0), *P* < 0.001]. The odds of clinical signs of pSBI were increased for all clinical signs except convulsions, for which no difference in association was found and it was not included in the final multivariable model (Table S4, Supplemental Digital Content 1, http://links.lww.com/INF/C774).

#### 5. Neonates With Clinically Nonsignificant or Uncertain Significance Isolates (Excluding CoNS) Compared with all Other Admissions

In neonates with other presumed clinically nonsignificant pathogens or isolates of unknown significance from blood culture in this context (*Streptococcus viridans*, Coryneforms, *Micrococcos* spp. and *Bacillus* spp.), there was no association with death compared with all other admissions [OR = 1.2 (1.0–1.5), *P* = 0.1) nor increased duration of admission [OR = 1.1 (0.9–1.3), *P*=0.5] nor association with clinical signs of pSBI (Table S5, Supplemental Digital Content 1, http://links.lww.com/INF/C774).

## DISCUSSION

CoNS was frequently isolated from neonates admitted to KCH (~1 in 10). However, there was no evidence for an association with inpatient death or prolonged duration of admission compared with other admissions. This was despite empiric treatment without a specific anti-Staphylococcal agent, although gentamicin may offer some cover for methicillin-sensitive isolates,^32^ suggesting that current treatment strategies do not need to be redirected to cover CoNS in this setting.

However, we cannot exclude that in a small number of individual cases, CoNS may have been clinically significant, because the frequent isolation of CoNS, in 44% of positive blood cultures, may introduce sufficient misclassification that associations with adverse outcomes from true CoNS infections are undetectable. In addition, CoNS was associated with convulsions. It is possible that there is a true association between CoNS infection and convulsions, and investigation of CSF would be helpful to examine this. However, the association with convulsions may be a chance result or by confounding because it is possible that convulsions make it more difficult for a blood sample to be obtained and increase skin contamination. This was not accounted for by small blood volumes alone, which was adjusted for, but, for example, from movement during the procedure.

While the systematic collection of clinical and microbiologic data at admission is a strength of the study, as CoNS is more likely to act as a pathogen after admission (in association with indwelling devices) and here almost all sampling was done within 48 hours of admission, it is possible that neonatal blood stream infection with CoNS occurring after admission could be systematically underrepresented.

However, the results of this study, suggest the majority of CoNS isolations were not clinically significant, implying that with 44% of all positive neonatal blood cultures isolating CoNS, contamination was frequent. This is higher than the 6%–32% of all positive blood cultures reported in Zambia and Ghana.^13,15,24^ This comparison should be made with caution, however, because these studies did not sample all neonatal admissions, and those most difficult to sample, and perhaps most likely to have CoNS isolated, would not, therefore, have been included. However, the introduction of quality improvement activity from 2009 did appear to reduce isolation of CoNS, and other organisms of uncertain clinical significance, suggesting that contamination can be reduced.

The findings of the study are generalizable to the majority of health care facilities in sub-Saharan Africa with similar levels of care, where for example, indwelling devices are mostly limited to peripheral cannulas. There are, however, tertiary centers in Kenya,^33^ and other middle-income countries in sub-Saharan Africa such as South Africa,^34^ where the use of indwelling devices and ventilation are common practice and where findings may differ. CoNS may yet emerge as an important pathogen in resource-poor settings as more invasive techniques to support neonatal care are used. To ensure clinical practice is based on the most up-to-date and relevant data, it is essential that long-term surveillance of serious bacterial neonatal infections is increased at all levels of health care in sub-Saharan Africa. In addition, if studies consistently report all blood stream isolates (whether regarded as clinically significant or not),^35^ we will increase our understanding of the etiology of neonatal sepsis in different settings, especially if contamination at the time of sampling can be reduced.

Our findings suggest that neonatal CoNS bacteremia is not associated with adverse clinical outcomes when neonates are treated according to standard WHO guidelines.

However, improving clinical surveillance in health care facility settings to inform case management is essential, to continue to direct treatment guidelines and inform changes, when needed, to local, national and international policies.

## ACKNOWLEDGMENTS

This study is published with the permission of the Director of Kenya Medical Research Institute. We thank the KEMRI-Wellcome Trust Research Programme.

## Supplementary Material
