# Supplementary material for: Should First-line Empiric Treatment Strategies for Neonates Cover Coagulase-negative Staphylococcal Infections in Kenya?
Source: Pediatr Infect Dis J. 2017 Oct 13;36(11):1073–8. doi: 10.1097/INF.0000000000001699 (PMC5640986; doi:10.1097/INF.0000000000001699)
Supplement: Supplementary file 1 [file inf-36-1073-s001.pdf]

Supplementary Digital Content

Should first-line empiric treatment strategies for neonates cover coagulase-negative Staphylococcal infections in Kenya?

Anna C. Seale DPhil,*† §, Christina W. Obiero, MBChB, MPH,*† Kelsey Jones, PhD,* ‡ Hellen C. Barsosio MBChB*, Johnstone Thitiri, MSc,* Moses Ngari, MSc,* Susan Morpeth, PhD,*§ Shebe Mohammed, HND,* Greg Fegan, PhD,*† Neema Mturi, MRCP,* James A. Berkley, FRCPCH,*†

*KEMRI-Wellcome Trust Research Institution, Kilifi, Kenya; †University of Oxford, UK; ‡Imperial College London, London, UK; §London School of Hygiene and Tropical Medicine, London, UK

Contents

Table S1: Characteristics of neonates admitted with clinically significant bacteraemias* from Kilifi County Hospital 1998-2013 3

Table S2 Demographic and clinical characteristics associated with CoNS isolation in the blood, in neonates admitted to Kilifi County Hospital 1998-2013 compared to neonates with no clinically significant isolate (or no growth). 4

Table S3 Demographic and clinical characteristics associated with CoNS isolation in the blood, in neonates admitted to Kilifi County Hospital 1998-2013 compared to neonates with a clinically significant bacteraemia 5

Tables S4 Demographic and clinical characteristics associated with neonates admitted with clinically significant bacteraemia admitted to Kilifi County Hospital 1998-2013 6

Table S5 Demographic and clinical characteristics associated with isolation of clinically non-significant isolates from blood in neonates admitted to Kilifi County Hospital 1998-2013 7

Figure S1 Percentage of blood culture isolations by type per year in neonates admitted to Kilifi County Hospital 1998-2013 8

Table S1: Characteristics of neonates admitted with clinically significant bacteraemias* from Kilifi County Hospital 1998-2013

		Total	Pathogen N= 689	%
Age	<2 days	4283	185	26.9
	2-6 days	2154	226	32.8
	7-13 days	1863	156	22.6
	14-20 days	747	79	11.5
	21-27 days	505	43	6.2
Sex	Female	4033	287	41.7
	Male	5519	402	58.3
Weight	<1500g	1318	124	18.0
	1500g <2500g	2819	205	29.8
	2500g <3500g	4393	286	41.5
	≥3500g	844	61	8.9
	Missing	178	13	1.9
HIV	Negative	4388	231	33.5
	Positive	225	24	3.5
	No consent	20	3	0.4
	Period not tested	4265	401	58.2
	Missing	654	30	4.4
Difficulty feeding	No	8413	569	82.6
	Yes	1111	116	16.8
	Missing	28	4	0.6
High RR (>60)	No	6406	458	66.5
	Yes	2814	209	30.3
	Missing	332	22	3.2
Severe indrawing	No	5966	427	62.0
	Yes	3560	259	37.6
	Missing	26	3	0.4
Movement only when stimulated (lethargy)	No	7451	626	90.9
	Yes	661	61	8.9
	Missing	1440	2	0.3
Impaired conscious level	No	7165	464	67.3
	Yes	2361	223	32.4
	Missing	26	2	0.3
Convulsions	No	9043	645	93.6
	Yes	494	43	6.2
	Missing	15	1	0.1
Temperature	<35.5	3831	116	16.8
	35.5-37.5	3038	298	43.3
	>37.5	2580	262	38.0
	Missing	103	13	1.9
Died	No	7679	448	65.0
	Yes	1873	241	35.0
Duration of admission (days)	<7	4439	364	52.8
	7 <14	3240	325	47.2

*Includes: *E.Coli*, GBS, GAS, *Klebsiella pneumoniae, Staphylococcus aureus, Citrobacter sp, Salmonella sp., Aeromonas sp., Streptococcus pneumoniae, Plesiomonas shigelloides, Acinetobacter sp., Enterobacter cloacae, Proteus mirabilis, Vibrio sp, Pseudomonas sp.*

Table S2 Demographic and clinical characteristics associated with CoNS isolation in the blood, in neonates admitted to Kilifi County Hospital 1998-2013 compared to neonates with *Bacillus sp., Coryneforms, Micrococcus* sp. and Viridans-group *Streptococci* (or no growth).

		Univariable †			Multivariable (8863) ††		
		OR	95%CI	p	OR	95%CI	p**
Age*	<2 days	1		<0.001	1		<0.001
	2-6 days	2.7	(2.3-3.3)		2.7	(2.3-3.3)	
	7-13 days	3.3	(2.8-4.0)		3.3	(2.8-4.0)	
	14-20 days	3.4	(2.6-4.3)		3.4	(2.6-4.3)	
	21-27 days	3.3	(2.4-4.3)		3.3	(2.4-4.3)	
Sex*	Female	1		0.066	1		0.052
	Male	1.1	(1.0-1.3)		1.1	(1.0-1.3)	
Difficulty feeding	No	1		0.7			
	Yes	1.0	(0.8-1.2)				
RR >60 bpm	No	1		0.3			
	Yes	0.9	(0.8-1.1)				
Movement only when stimulated	No	1		0.3			
	Yes	0.9	(0.7-1.2)				
Severe indrawing	No	1		0.063	1		0.088
	Yes	0.9	(0.7-1.0)		0.9	(0.7-1.0)	
Impaired conscious level	No	1		0.1			
	Yes	0.9	(0.7-1.0)				
Convulsions	No	1		0.027	1		0.041
	Yes	1.4	(1.0-1.8)		1.3	(1.0-1.7)	
Temperature	<35.5	0.9	(0.7-1.1)	0.6			
	35.5-37.5	1					
	≥37.5	1.0	(0.9-1.2)				
Blood Volume*	Baseline	1		<0.001	1		<0.001
	Per mL increase	0.8	(0.7-0.9))		0.8	(0.7-0.9)	

*a priori confounders
**from the likelihood ratio test
† adjusted for age, sex and blood volume
† adjusted for age, sex and blood volume and clinical signs associated in univariable analyses

Table S3 Demographic and clinical characteristics associated with CoNS isolation in the blood, in neonates admitted to Kilifi County Hospital 1998-2013 compared to neonates with a clinically significant bacteraemia

		Univariable †			Multivariable (8863) ††		
		OR	95%CI	p	OR	95%CI	p**
Age*	<2 days	1		0.026	1		0.053
	2-6 days	0.9	(0.7-1.2)		0.8	(0.6-1.1)	
	7-13 days	1.4	(1.1-1.9)		1.2	(0.9-1.6)	
	14-20 days	1.1	(0.8-1.6)		1.0	(0.7-1.4)	
	21-27 days	1.3	(0.9-2.0)		1.2	(0.7-1.9)	
Sex*	Female	1		0.6	1		0.5
	Male	1.1	(0.9-1.3)		1.1	(0.9-1.3)	
Difficulty feeding	No	1		0.001			
	Yes	0.6	(0.5-0.8)				
RR >60 bpm	No	1		0.045			
	Yes	0.8	(0.6-1.0)				
Movement only when stimulated	No	1		0.025	1		0.003
	Yes	0.6	(0.4-0.9)		0.5	(0.4-0.8)	
Severe indrawing	No	1		<0.001	1		0.026
	Yes	0.7	(0.6-0.9)		0.8	(0.6-1.0)	
Impaired conscious level	No	1		<0.001	1		<0.001
	Yes	0.5	(0.4-0.6)		0.5	(0.4-0.6)	
Convulsions	No	1		0.5			
	Yes	1.1	(0.8-1.7)				
Temperature	<35.5	0.6	(0.4-0.8)	<0.001	0.7	(0.5-0.9)	<0.001
	35.5-37.5	1			1		
	≥37.5	0.6	(0.5-0.8)		0.7	(0.5-0.8)	
Blood Volume*	Baseline	1		0.075	1		0.2
	Per mL increase	0.9	(0.7-1.0)		0.8	(0.7-0.9)	

*a priori confounders
**from the likelihood ratio test
† adjusted for age, sex and blood volume
† adjusted for age, sex and blood volume and clinical signs associated in univariable analyses

Tables S4 Demographic and clinical characteristics associated with neonates admitted with clinically significant bacteraemia admitted to Kilifi County Hospital 1998-2013 compared to all other admissions

		Univariable †			Multivariable (9063) ††		
		OR	95%CI	p	OR	95%CI	p**
Age*	<2 days	1		<0.001	1		<0.001
	2-6 days	2.7	(2.2-3.3)		3.3	(2.6-4.2)	
	7-13 days	2.1	(1.7-2.6)		2.5	(1.9-3.3)	
	14-20 days	2.6	(2.0-3.5)		3.2	(2.4-4.4)	
	21-27 days	2.2	(1.5-3.1)		2.5	(1.7-3.7)	
Sex*	Female	1		0.5	1		0.5
	Male	1.1	(0.9-1.3)		1.1	(0.9-1.3)	
Difficulty feeding	No	1		<0.001	1		0.049
	Yes	1.6	(1.3-2.0)		1.3	(1.0-1.6)	
RR >60 bpm	No	1		0.060			
	Yes	1.2	(1.0-1.4)				
Movement only when stimulated	No	1		0.010	1		<0.001
	Yes	1.5	(1.1-1.9)		1.7	(1.3-2.3)	
Severe indrawing	No	1		0.004	1		0.029
	Yes	1.3	(1.1-1.5)		1.2	(1.0-1.5)	
Impaired conscious level	No	1		<0.001	1		<0.001
	Yes	1.8	(1.5-2.2)		1.7	(1.4-2.1)	
Convulsions	No	1		0.5			
	Yes	1.1	(0.8-1.6)				
Temperature	<35.5	1.6	(1.3-2.1)	<0.001	1.4	(1.1-1.8)	<0.001
	35.5-37.5	1			1		
	≥37.5	1.5	(1.3-1.8)		1.5	(1.3-1.8)	
Blood Volume*	Baseline	1		0.6	1		0.3
	Per mL increase	1.0	(0.9-1.3)		0.9	(0.8-1.1)	

*a priori confounders
**from the likelihood ratio test
† adjusted for age, sex and blood volume
† adjusted for age, sex and blood volume and clinical signs associated in univariable analyses

Table S5 Demographic and clinical characteristics associated with isolation of *Bacillus* sp., Coryneforms, *Micrococcus* sp. and Viridans-group *Streptococci* from blood in neonates admitted to Kilifi County Hospital 1998-2013 compared to all other admissions

		Univariable †			Multivariable (9063) ††		
		OR	95%CI	p	OR	95%CI	p**
Age*	<2 days	1		<0.001	1		<0.001
	2-6 days	1.8	(1.4-2.2)		1.8	(1.4-2.2)	
	7-13 days	1.6	(1.3-2.0)		1.6	(1.2-2.0)	
	14-20 days	1.0	(0.7-1.4)		1.0	(0.6-1.4)	
	21-27 days	1.4	(0.9-2.0)		1.3	(0.9-2.0)	
Sex*	Female	1		0.027	1		0.028
	Male	1.2	(1.0-1.5)		1.2	(1.0-1.5)	
Difficulty feeding	No	1		0.8			
	Yes	1.0	(0.8-1.4)				
RR >60 bpm	No	1		0.8			
	Yes	1.0	(0.8-1.2)				
Movement only when stimulated	No	1		0.3			
	Yes	1.2	(0.8-1.6)				
Severe indrawing	No	1		0.7			
	Yes	1.0	(0.9-1.3)				
Impaired conscious level	No	1		0.086	1		0.085
	Yes	0.8	(0.7-1.0)		0.8	(0.7-1.0)	
Convulsions	No	1		0.3			
	Yes	1.2	(0.8-1.7)				
Temperature	<35.5	0.9	(0.7-1.2)	0.6			
	35.5-37.5	1					
	≥37.5	0.9	(0.7-1.2)				
Blood Volume*	Baseline	1		<0.001	1		0.004
	Per mL increase	0.8	(0.7-0.9))		0.8	(0.7-0.9)	

*a priori confounders
**from the likelihood ratio test
† adjusted for age, sex and blood volume
† adjusted for age, sex and blood volume and clinical signs associated in univariable analyses

Figure S1 Percentage of blood culture isolations by type per year in neonates admitted to Kilifi County Hospital 1998-2013

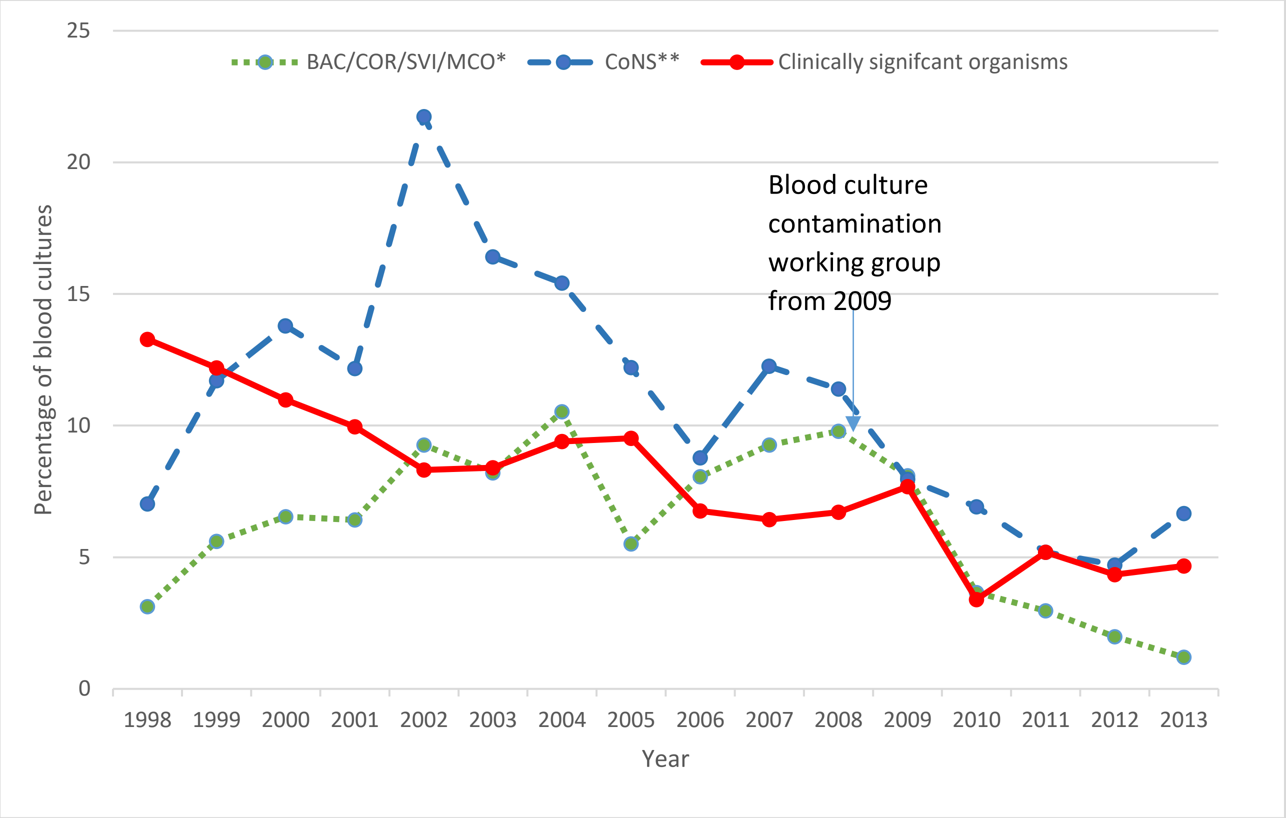

*BAC= *Bacillus* sp.; COR= Coryneforms; SVI= Viridans-group *Streptococci;* MCO= *Micrococcus* sp.
**CoNS= Coagulase negative Staphylococci
